# Long-term effects of mobile exoneuromusculoskeleton (ENMS)-assisted self-help telerehabilitation after stroke

**DOI:** 10.3389/fnins.2024.1371319

**Published:** 2024-03-13

**Authors:** Wanyi Qing, Ching-Yi Nam, Harvey Man-Hok Shum, Marko Ka-Leung Chan, King-Pong Yu, Serena Sin-Wah Ng, Bibo Yang, Xiaoling Hu

**Affiliations:** ^1^Department of Biomedical Engineering, Research Institute for Smart Ageing (RISA), The Hong Kong Polytechnic University, Kowloon, Hong Kong SAR, China; ^2^Community Rehabilitation Service Support Centre, Queen Elizabeth Hospital, Kowloon, Hong Kong SAR, China; ^3^Research Centre of Data Science and Artificial Intelligence (RC-DSAI), The Hong Kong Polytechnic University, Kowloon, Hong Kong SAR, China; ^4^Joint Research Centre for Biosensing and Precision Theranostics, The Hong Kong Polytechnic University, Kowloon, Hong Kong SAR, China; ^5^University Research Facility in Behavioral and Systems Neuroscience (UBSN), The Hong Kong Polytechnic University, Kowloon, Hong Kong SAR, China

**Keywords:** stroke, upper limb, robot, rehabilitation, telerehabilitation, long term

## Abstract

Investigation on long-term effects of robot-assisted poststroke rehabilitation is challenging because of the difficulties in administration and follow-up of individuals throughout the process. A mobile hybrid neuromuscular electrical stimulation (NMES)-robot, i.e., exoneuromusculoskeleton (ENSM) was adopted for a single-group trial to investigate the long-term effects of the robot-assisted self-help telerehabilitation on upper limb motor function after stroke. Twenty-two patients with chronic stroke were recruited to attend a 20-session telerehabilitation program assisted by the wrist/hand module of the ENMS (WH-ENMS). Participants were evaluated before, after, as well as at 3 months and 6 months after the training. The primary outcome measure was the Fugl-Meyer Assessment-Upper Extremity (FMA-UE), supplemented by secondary outcome measures of the FMA-UE of the shoulder and elbow (FMA shoulder/elbow), the FMA-UE of the wrist and hand (FMA wrist/hand), the Modified Ashworth Scale (MAS), the Action Research Arm Test (ARAT), the Wolf Motor Function Test (WMFT), the Functional Independence Measure (FIM), as well as electromyography (EMG) and kinematic measurements. Twenty participants completed the telerehabilitation program, with 19 returning for a 3-month follow-up, and 18 for a 6-month follow-up. Significantly improved clinical scores were observed after the training (*p* ≤ 0.05). These improvements were maintained after 6 months in the FMA-UE, FMA shoulder/elbow, MAS at the wrist flexor, WMFT score, WMFT time, and FIM (*p* ≤ 0.05). The maintained improvements in motor function were attributed to reduced muscular compensation, as indicated by EMG and kinematic parameters. The WH-ENMS-assisted self-help telerehabilitation could achieve long-lasting rehabilitative effects in chronic stroke.

## 1 Introduction

Stroke has become the main reason for acquired adult disability worldwide, with 85% sustained residual impairments in the upper limb after being discharged home, especially in the distal joints of the upper limb (i.e., the wrist and hand) because of a delayed restoration compared to the proximal joints (i.e., shoulder and elbow) (Chen and Winstein, 2009; Martino Cinnera et al., 2024). It has been reported that repetitive and intensive practice is effective for upper limb motor restoration even in the chronic stage after stroke (Donnellan-Fernandez et al., 2022). However, most discharged patients could not gain access to wrist-hand services because much more rehabilitation resources are devoted to the subacute stage due to a shortage of professional manpower (Jesus et al., 2017). Recently, rehabilitation robots have been used to complement conventional therapy with the advantage of facilitating repetitive movement training with high intensity when professional manpower is insufficient (Morone et al., 2020). The therapeutic effects of robot-assisted intervention have been demonstrated previously, which was comparable and even superior to conventional treatment in motor improvements of the upper limb (Zhang et al., 2022).

Home-based telerehabilitation with minimal assistance and remote supervision could be an augmentation to public clinical service. In contrast to traditional center-based therapy, conducting robot-assisted training within the home environment may promote more effective integration of training routines into daily life over the longer term with advantages of reducing traffic and healthcare burden (Khan et al., 2023). However, the preparation and management of telerehabilitation is challenging for real clinical practice, which might influence the training effects (Qing et al., 2023). On the other hand, the focus of most clinical trials has been on the immediate outcomes after intervention, with limited attention given to the long-term effects of robot-assisted telerehabilitation during follow-ups. The challenges associated with conducting the long-term follow-up are noteworthy, particularly due to high drop-out rates observed in individuals with unstable neurological conditions such as stroke, as well as subjects left the clinical trial sites, which contributed to potentially adverse biases on the results (Desai, 2020). Rare reports have covered the long-term effects of robot-assisted poststroke intervention, particularly for chronic stroke. However, it is crucial to recognize that the long-term effects of robot-assisted telerehabilitation are clinically important for evaluating the stabilization and generalization of the treatment effects, considering the distinct changes in motor and activity function observed at the end of treatment compared to those observed during follow-ups. A previous study (Burgar et al., 2011) revealed that notable differences were found in the gains achieved in the Fugl-Meyer Assessment-Upper Extremity (FMA-UE) and the Functional Independence Measure (FIM) between the robotic and control groups at discharge but these differences were not sustained at the six-month follow-up. Consequently, the duration, for which the effects of robot-assisted telerehabilitation persist, remains inconclusive. Moreover, these studies mainly relied on subjective clinical assessments, which are experience-related and lack objectivity and precision, with limited utilization of quantitative evaluations such as electromyography (EMG) and kinematic assessments. Further investigation is warranted to elucidate the muscular and kinematic changes following robot-assisted telerehabilitation.

In our previous work, a mobile exoneuromusculoskeleton (ENMS) was designed for poststroke upper limb rehabilitation (Nam et al., 2022). The system integrated neuromuscular electrical stimulation (NMES) and robot in one mechatronic platform, driven by voluntary efforts from the elbow, wrist, and finger. We translated a self-help telerehabilitation program assisted by the wrist-hand model of the ENMS (WH-ENMS) from laboratory research to the routine practice of a local public rehabilitation center for validation of the feasibility with short-term outcomes (Qing et al., 2023). Twelve participants with chronic stroke completed the training according to the protocol and obtained significant motor improvements in the upper limb without adverse or safety reports (Qing et al., 2023). Meanwhile, we continued the telerehabilitation program and recruited a total of 22 persons with chronic stroke. The study’s purpose was to investigate the long-term effects extending to 6 months after the WH-ENMS-assisted telerehabilitation in terms of clinical scores, muscular improvements, and kinematic performance.

## 2 Method

This study employed a pre-post single group design with follow-up assessments at 3 months (3MFU) and 6 months (6MFU). The study involved 20 sessions of home wrist-hand training using the WH-ENMS for stroke survivors with residual upper limb impairments. Before the experiment, approval was acquired from the Human Participants Ethics Sub-Committee of the Hong Kong Polytechnic University and Research Ethics Committee (Kowloon Central and Kowloon East).

### 2.1 EMG-driven WH-ENMS

The WH-ENMS is a hybrid system that integrates pneumatic actuation with NMES driven by residual EMG from the paretic upper extremity after a stroke, assisting a stroke survivor to complete repetitive, phased, and coordinated wrist and hand movements, such as (1) wrist extension with the hand open and (2) wrist flexion with the hand closed (Nam et al., 2022). Components of the WH-ENMS include a glove embedding pneumatic fingers and a three-dimensional printed exoskeletal connector, two channels of EMG-NMES, a pump and control box with a rechargeable 12-V battery, and a matched smartphone application (App) (Nam et al., 2022). The developed App communicates with the control box wirelessly via Bluetooth, providing a friendly interface for easy operation. The extensor carpi ulnaris (ECU) and extensor digitorum (ED), and the flexor carpi radialis (FCR) and flexor digitorum (FD), were considered as two muscle unions (i.e., ECU-ED and FCR-FD) because of the proximity of their anatomical locations. The WH-ENMS adopted EMG-triggered control using ECU-ED and FCR-FD as voluntary neuromuscular drives to initiate mechanical and NMES assistances in each motion phase. Two channels of EMG-NMES were used to detect EMG signals and deliver the NMES through two pairs of reusable surface electrodes placed on the common area of muscle bellies’ motor points of the ECU-ED and FCR-FD (Nam et al., 2021). In the “wrist extension with the hand open” phase, once the EMG activation level of the ECU-ED reached the preset threshold, the pneumatic fingers inflated to provide the extension torque to each digit. In the “wrist flexion with the hand closed” phase, once the EMG activation level of the FCR-FD reached the preset threshold, the pneumatic fingers deflated passively due to the voluntary finger flexion of the paretic limb. The NMES was used to assist wrist extension with the hand open via ECU-ED and wrist flexion with the hand closed via FCR-FD. A reference electrode was attached to the skin surface of the olecranon to reduce the common-mode noise.

### 2.2 Participants

Previously, we translated a telerehabilitation program to the Community Rehabilitation Service Support Centre of the Hospital Authority (CRSSC) in Hong Kong (Qing et al., 2023). In this scenario, we kept recruiting participants from outpatients admitted to the CRSSC in the period from April 2020 to March 2023. Patients were included if they had: (1) single and unilateral brain damage from a stroke that occurred longer than 12 months; (2) Modified Ashworth Scale (MAS) score less than 3 at the elbow, wrist, and finger (Ashworth, 1964); (3) FMA-UE score over 15 (Fugl-Meyer et al., 1975); (4) Mini-Mental State Examination score over 21 (Folstein et al., 1975); (5) detectable voluntary EMG signals of the driving muscles (ECU-ED, FCR-FD) on the paretic side (i.e., three times the standard deviation (SD) above the EMG baseline); (6) FIM of at least 51; (7) fulfillment of the minimal requirements in the home, including a bridge chair without wheels, a table with a minimum surface area of 60 × 40 cm^2^ for the training space, and a 3G or above mobile network access. Patients were excluded if they (1) were epileptic, (2) had a cardiac pacemaker or other implants (e.g., neural implants), (3) had open wounds or skin lesions around the driving muscles, (4) had acute inflammation, (5) had shoulder pain, or poststroke central pain conditions, (6) had other neurological impairments besides stroke, or (7) were receiving other upper limb treatments during the telerehabilitation program period.

### 2.3 Training protocol

**Figure 1:**
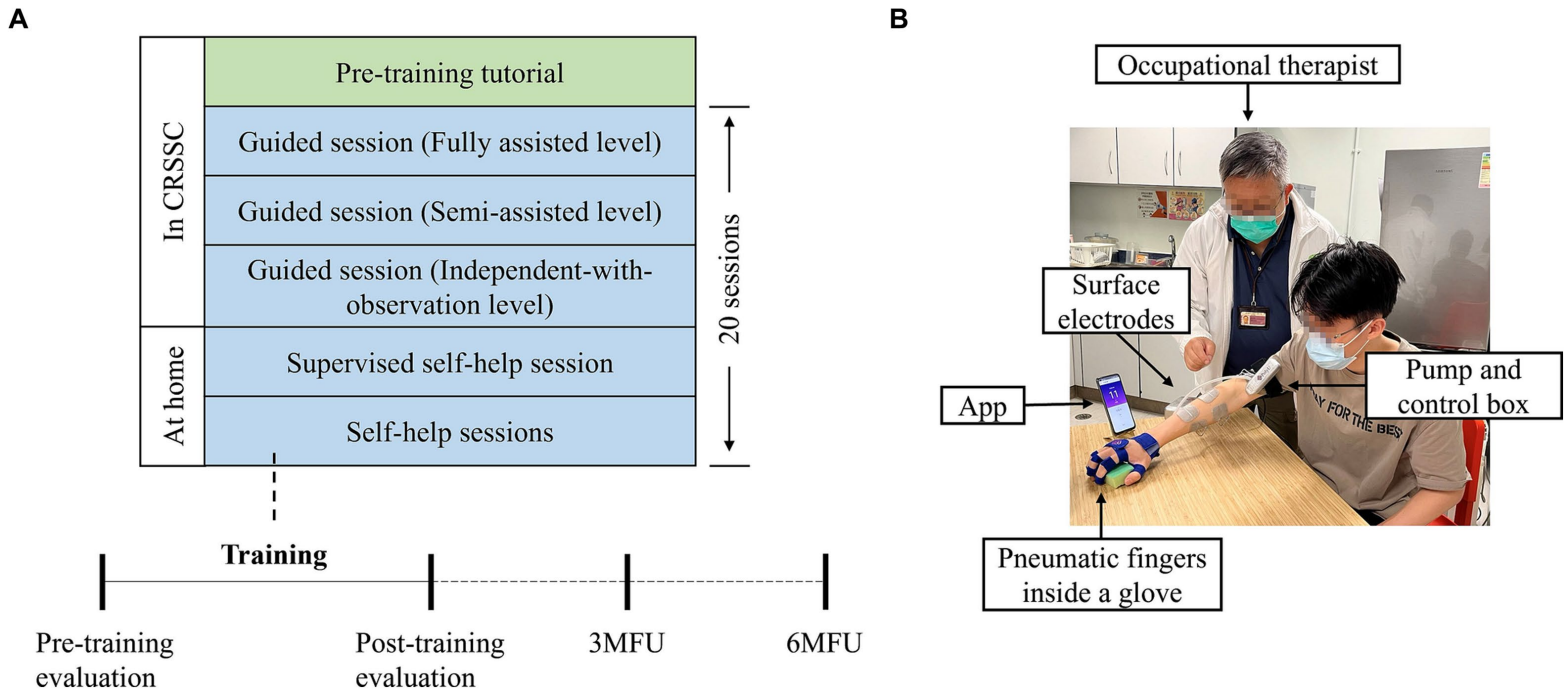
Overview of the WH-ENMS assisted telerehabilitation program. **(A)** Timeline for the 20-session telerehabilitation program. **(B)** The first self-help session supervised by the occupational therapist at home.

The telerehabilitation program consisted of 20-session WH-ENMS assisted upper limb training, including three guided sessions in the clinic setting and remaining sessions at home, with an intensity of 60–90 min per session, 3–5 sessions/week, 1 session/day at most, and completed in 7 consecutive weeks (Figure 1). Remote and professional supervision on training progresses based on the automatic feedback from the WH-ENMS, e.g., start/end times of training and repetitions of wrist-hand tasks, was provided in the home sessions.

The first three sessions and a pre-training tutorial (i.e., mandatory courses) were provided to persons after stroke, and/or their caregivers, under on-site professional assistance and supervision for preparation of the following self-help sessions at home. The experimental operators were a CRSSC-employed, registered occupational therapist and an occupational therapy assistant. Mandatory courses were conducted in an independent training room at the CRSSC. Prior to the training, each participant and/or their caregivers were provided with an introductory tutorial on donning and doffing the system, system operation, and the training protocols (Nam et al., 2021). Training parameters, including the EMG threshold level, NMES assistance level, and mechanical assistance level, were set by the experimental operator, and those parameters remained fixed throughout the 20 sessions of training for a participant. If a participant did not have a smartphone, the experimental operator lent him/her one until they completed the program. After the pre-training tutorial, three guided training sessions were provided to each participant. In each training session, participants were asked to sit by a table and maintain their shoulders above the table with a vertical distance of 30–40 cm. A smartphone with an App to provide visual clues during the training was placed on the table at a distance of 30–60 cm in front of the participant. Participants were required to perform repetitive limb tasks during the training. The repetitive limb tasks included a horizontal task, a vertical task, and an optional forward task (Qing et al., 2023). The horizontal task referred to gripping a sponge (8.5 × 5.5 cm^2^) from the participant’s affected side, dropping it laterally 50 cm away on the other side, and then returning it to the initial location. The vertical task referred to gripping a sponge from underneath an 18-cm-high shelf to the top of the shelf and then returning it to its initial location. The forward task referred to gripping a sponge at a forward distance of 30 cm and returning it to its initial location. Each limb task was required to be repeated for 30 min. Thus, the duration of each training session ranged from 60 to 90 min. A 10-min rest was allowed between the two consecutive tasks to prevent muscle fatigue. The three guided training sessions were supervised and assisted by the experimental operator. In the guided training sessions, onsite feedback by the operators was provided to enhance participants’ performance and to support their transition to self-help training at home. If the experimental operator deemed a participant competent to perform the self-help training, they were required to conduct the remaining sessions at home. In the first self-help session, the experimental operator delivered the WH-ENMS with a charger and training props (i.e., a sponge and a shelf) to the participant’s home, inspected the safety of the home, and observed a training session to ensure consistency with the onsite sessions. Each participant was required to pay HKD375 for the mandatory courses at the CRSSC, as per the routine management. A detailed description of the experimental protocol has been reported in our previous study (Qing et al., 2023).

Training data, including the training frequency, session duration, and complete movement cycles, were recorded by the developed App and automatically transmitted to a server in the neurorehabilitation lab at the university through a 3G or above mobile network after each session. Experimental operators remotely monitored the training data of each self-help session based on a pre-set training schedule agreed on by the participant before the training. If the participant missed a session, the experimental operator contacted the participant by telephone or message to arrange a make-up session according to the protocol training intensity. If a participant encountered any technical problems with the WH-ENMS at home, they were required to report it to the experiment operator immediately by telephone or message. A backup system was prepared for each participant before the training started and stored at the CRSSC for replacement. A malfunctioning system was replaced within a working day to avoid a violation of the training protocol. The malfunctioning system was returned to the research team for inspection and replacement. Additionally, the experimental operator contacted the ongoing participants weekly via telephone or message to discuss their experiences.

### 2.4 Evaluation

Participants were evaluated before the pre-training tutorial (i.e., the pre-training evaluation), the day after the last training session (i.e., the post-training evaluation), 3 months after the training (i.e., 3MFU), 6 months after the training (i.e., 6MFU) (Figure 1A). To ensure the stability of the baseline, clinical evaluations were carried out three times within 2 weeks before the training as the baseline, with a minimum two-day interval between the evaluations. For the statistical calculations, the mean of the three pre-training evaluations was used. The EMG evaluations and kinematic evaluations were conducted at four time points (i.e., the pre-training evaluation, the post-training evaluation, 3MFU, and 6MFU) for objective measurements of the muscular coordination and kinematic performance of the paretic upper limb. The primary outcome of this study was the FMA-UE. The other clinical scores, EMG parameters, and kinematic parameters were secondary outcomes.

The clinical evaluation was conducted by an assessor who was blinded to the protocol. Clinical measures included (1) the FMA-UE, which is a 66-score scale divided into 42 scores for the FMA-UE of the shoulder and elbow (FMA shoulder/elbow) and 24 scores for the FMA-UE of the wrist and hand (FMA wrist/hand). It is considered a reliable measure to detect the motor function improvement of the upper limb with robotic training (Fugl-Meyer et al., 1975; Wei et al., 2011); (2) MAS at the elbow, wrist, and finger flexors, which is the most widely used scale to evaluate muscle tone (Ashworth, 1964; Gregson et al., 1999); (3) the Action Research Arm Test (ARAT), which evaluates the proximal and distal arm motor function (Lyle, 1981); (4) the Wolf Motor Function Test (WMFT), which comprises of 17 tasks and records the score and time it takes to complete each task (Wolf et al., 1989); (5) the FIM, which is generally used to measure the disability degree in daily living (Mackintosh, 2009).

EMG signals from the ECU-ED, abductor pollicis brevis (APB), triceps brachii (TRI), biceps brachii (BIC), and FCR-FD muscles of the paretic upper extremities were recorded to quantitatively measure muscles’ activation and coordination. During an EMG evaluation, maximum voluntary contractions (MVCs) of each muscle were first acquired, followed by bare-arm tests, which are the same as horizontal and vertical tasks during the training without wearing the WH-ENMS with a repetition of three times. A two-minute rest period was provided between two consecutive contractions to avoid muscle fatigue. The collected EMG signals were first amplified with a gain of 1,000 (amplifier: INA 333, Texas Instruments Inc., Dallas, TX, USA), band-pass filtered from 10 to 500 Hz, and then sampled at 1000 Hz. During the offline processing, the EMG was rectified and filtered by a 4th-order Butterworth lowpass filter with the cutoff at 10 Hz to obtain the envelope of an EMG signal trial. Two EMG parameters were used to analyze the training progress, i.e., the activation level of each target muscle and the EMG co-contraction index (CI) of muscle pairs (Hu et al., 2009). The activation level of a muscle was an averaged level of the EMG signal envelope with respect to its maximum value in MVCs. The EMG CI of a muscle pair evaluated the independence of the two muscles and was the overlapping parts in the EMG signal envelope of a muscle pair, as detailed in Qing et al. (2023).

Three-dimensional motions of the paretic upper limb were captured by a motion capture system (Vicon Motion Systems, Oxford, UK) to quantify the kinematic performance. The participants were required to perform the same bare-arm test as in the EMG evaluation with a repetition of three times for each task. There was a two-minute break between two consecutive contractions to prevent muscle fatigue. Two kinematic variables, the number of movement units (NMUs) and maximal trunk displacement (MTD), were adopted to evaluate the movement smoothness and compensatory trunk movement (Thrane et al., 2020). NMUs were the cumulated counts of signified change in the tangential velocity of the middle finger’s metacarpophalangeal join during the bare-arm tests. MTD was the maximal distance of the trunk displacements with respect to an initial location during the bare-arm tests.

### 2.5 Statistical analysis

The statistical analysis was performed by SPSS version 26 (IBM, Chicago, IL, USA). All outcomes were subjected to normality tests using the Shapiro–Wilk test (Shapiro et al., 1968). WMFT scores and MTD were normally distributed at all time points (*p* > 0.05) and thus a one-way repeated measures analysis of variance (ANOVA) with a Bonferroni *post hoc* test was adopted to compare them before, after, 3 months, and 6 months after the training. Otherwise, a Friedman test with a Wilcoxon signed rank *post hoc* test was performed (Kim, 2014). The Bonferroni method was performed for the multiple comparison correction. *p* ≤ 0.05 was used as the statistically significant level for rejecting the null hypothesis for all analyses. The significance levels of *p* ≤ 0.01 and 0.001 were also indicated.

## 3 Results

Thirty-one outpatients admitted to the CRSSC were screened, and 22 were recruited to attend the training in a clinic setting. All participants gave written informed consent before the program started. However, two participants dropped out during the training because of personal reasons. A total of 20 participants completed the telerehabilitation program assisted by the WH-ENMS. Among them, 19 participants returned for a 3MFU evaluation, and 18 participants completed a 6MFU evaluation. The finally recruited 20 participants, including 13 males and 7 females, had an average age of 49.75 ± 13.13 (mean ± SD) years and average time since the onset of stroke of 3.71 ± 3.11 (mean ± SD) years. Eleven of them were left hemiplegia, and the other were right hemiplegia; and 6 had ischemic stroke, and 14 had hemorrhagic stroke. The average training frequencies of the participants were 3.45 ± 0.67 (mean ± SD) sessions per week. The average training durations per session were 89.80 ± 11.40 (mean ± SD) minutes per session. The average cycles of the completed movement were 173.02 ± 33.62 (mean ± SD) cycles per session.

**Figure 2:**
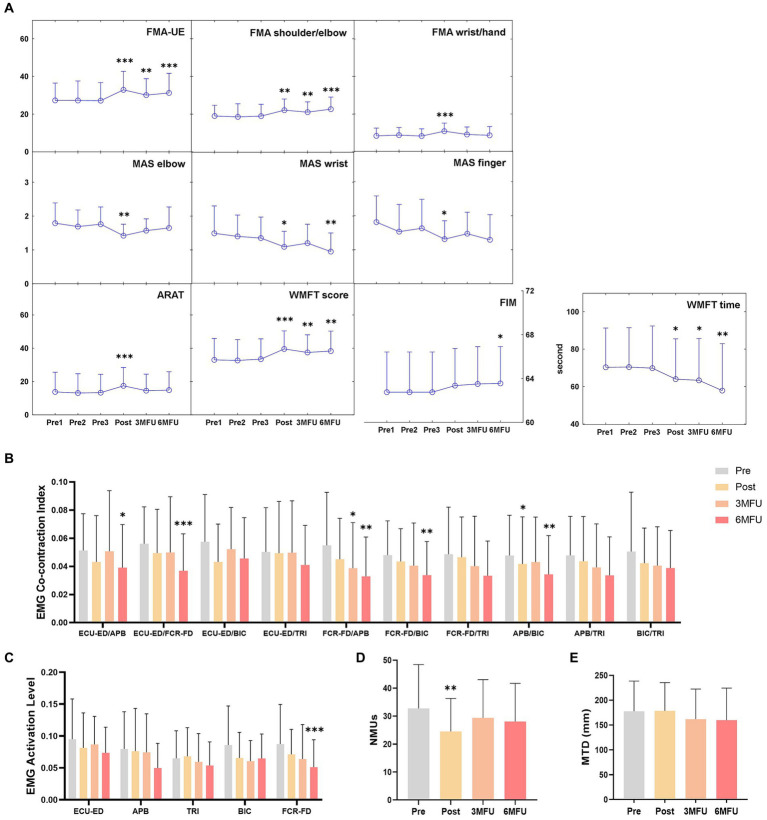
Longitudinal assessments on motor functions across different time points. **(A)** Clinical scores, **(B)** normalized EMG co-contraction index, **(C)** normalized EMG activation levels, **(D)** NMUs and **(E)** MTD before, after, 3 months and 6 months after the training represented by means and SDs. Significant levels are indicated by * (*p* ≤ 0.05), ** (*p* ≤ 0.01), and *** (*p* ≤ 0.001) for one-way repeated measures ANOVA with Bonferroni *post hoc* tests or Friedman tests with Wilcoxon signed rank *post hoc* tests.

Figure 2A illustrates the measured clinical scores before, after, 3 months, and 6 months after the training. Significant increases were observed in the FMA-UE, FMA shoulder/elbow, and FMA wrist/hand after the training (*p* ≤ 0.05). The increase in the FMA-UE and FMA shoulder/elbow were maintained at 3MFU and 6 MFU (*p* ≤ 0.05). There were significant decreases in the MAS at the elbow, wrist, and finger after the training (*p* ≤ 0.05); these decreases were kept for 6 months in the MAS at the wrist (*p* ≤ 0.05). In the ARAT, a significant increase was observed after the training (*p* ≤ 0.05). A significant increase in the WMFT score and a significant decrease in the WMFT time were identified after the training and kept at 3MFU and 6MFU (*p* ≤ 0.05). FIM obtained a significant increase 6 months after the training (*p* ≤ 0.05). The comparison results of clinical scores were summarized in Supplementary Table 1.

Figures 2B–E illustrates the EMG CI values, EMG activation levels, NMUs, and MTD before, after, 3 months, and 6 months after the training. There were significant decreases in the CI value of APB/BIC muscle pair after the training, CI value of FCR-FD/APB muscle pair at 3MFU, CI values of ECU-ED/APB, ECU-ED/FCR-FD, FCR-FD/APB, FCR-FD/BIC, APB/BIC muscle pairs at 6MFU compared with those before training (*p* ≤ 0.05). Among the EMG activation levels, a significant decrease was observed in the FCR-FD muscle at 6MFU (*p* ≤ 0.05). After the training, significant decrease was demonstrated in the NMUs (*p* ≤ 0.05). There was no significant difference in the MTD at any time point after the training. The comparison results of the EMG and kinematic parameters were summarized in Supplementary Table 2.

## 4 Discussion

### 4.1 Self-help WH-ENMS-assisted telerehabilitation resulted in long-term rehabilitative effects

In this single-group study, participants with chronic stroke completed the telerehabilitation program assisted by the WH-ENMS and achieved long-term improvements in the upper limb motor function validated by the follow-up evaluations. The telerehabilitation program could improve the motor function of the whole upper limb as indicated by the FMA-UE, FMA shoulder/elbow, FMA wrist/hand, and ARAT (Figure 2A). The enhancement in the motor function of the proximal upper limb could last to 3 months and 6 months after the training as indicated by the FMA shoulder/elbow (Figure 2A). Different from the previous studies of Nam et al. (2021) and Qing et al. (2023), activities of daily life (ADLs) were improved after the robot-assisted training. Actions close to ADLs, as indicated by WMFT score and WMFT time, ADLs, as indicated by FIM have improved for 6 months, which suggested functional generalization to daily activities (Figure 2A). Patients were anticipated to exhibit increased utilization of the affected upper limb in their daily activities after the intervention, owing to the enhancement of arm function. Some evidence suggests that following intense therapy, patients demonstrated heightened hand usage across various tasks, with these effects lasting up to 1 year (Rozevink et al., 2021). Moreover, conducting robot-assisted training in home environments showed advantages, as opposed to the traditional center-based therapy in clinical environments. The familiarity and comfort of home environments facilitated greater compliance of individual patients to a long-term training program which is fundamental to achieve motor restoration poststroke (Holmqvist and von Koch, 2001). On the other hand, the self-help rehabilitation at home promoted generalization of learned skills in daily activities, as indicated in the motor gains obtained in the 3 and 6-month follow-ups of this work. Therefore, integration of home-based rehabilitation as an augmentation to the traditional center-based and face-to-face clinical services may improve the holistic outcome of the rehabilitation.

### 4.2 Reduced muscular compensation and improved voluntary coordination in chronic stroke

In addition to clinical improvements, reduced muscular compensation has been observed during the long-term follow-up. Decreased EMG CI between muscle pairs, including ECU-ED/APB, ECU-ED/FCR-FD, FCR-FD/APB, FCR-FD/BIC, and APB/BIC, demonstrated improved independence of individual muscles with less compensation from others (Figure 2B). The decreased activation level of FCR-FD muscle over the 6 months indicated reduced muscle spasticity, which might be the reason for the decreased MAS at the wrist across the 6 months (Figures 2A, C). Decreased NMUs further supported improved movement smoothness and voluntary coordination after the training, aligning with the findings of EMG activation level and CI values between muscle pairs (Figure 2D). While this study demonstrated the maintenance of long-term training effectiveness for 6 months, it is crucial to note that not all robot-assisted training has exhibited enduring motor restoration in previous studies. For example, Burgar et al. (2011) reported postintervention effects that did not last 6 months. The long-lasting effects observed in our telerehabilitation program could be attributed to a better muscular coordinating pattern achieved in the training and generalized in the daily activities after the training. By integrating NMES and robot, the WH-ENMS used in this study could achieve close-to-normal muscular coordination with suppressed compensatory motions in the whole upper limb. As a result, it yielded better motor outcomes and faster recovery compared to those using robots or NMES only (Hu et al., 2015; Lee et al., 2015). Moreover, the WH-ENMS training required active engagement of distal upper limb muscles, i.e., FCR-FD and ECU-ED, which were typically compensated by the proximal upper limb muscles and exhibited poor muscular coordination (Jones, 2017). Participants actively triggered the system through voluntary motor efforts to complete the repetitive tasks, ensuring voluntary activation and reduced muscular compensation of target muscles in the rehabilitation process.

In the traditional outpatient service, therapists typically interact with patients on a one-to-one basis in face-to-face sessions. In this study, home-based telerehabilitation reduced the demand for on-site professional assistance and facilitated the training management of multiple patients at the same time. In future studies, we will quantify the cost-effectiveness of self-help telerehabilitation in large-scale clinical trials, compared to the traditional service. The quality of the home-based training by non-professionals was mainly affected by the operational skill of the device and the compliance with the training protocol. In this work, all participants and their caregivers grasped the skill in the guided training sessions before the remote self-help sessions. The adherence to the protocol of a participant during the home-based training was monitored by the professional remotely through the WH-ENMS log data (Qing et al., 2023). All participants of the work completed the training tasks assigned in the protocol.

## Data availability statement

The original contributions presented in the study are included in the article/Supplementary material, further inquiries can be directed to the corresponding author.

## Ethics statement

The studies involving humans were approved by The Human Participants Ethics Sub-Committee of the Hong Kong Polytechnic University (Ref: HSEARS20200304001) and Research Ethics Committee (Kowloon Central and Kowloon East, Ref: KC/KE-20-0114/FR-2). The studies were conducted in accordance with the local legislation and institutional requirements. The participants provided their written informed consent to participate in this study. ClinicalTrials.gov Register Number NCT03752775.

## Author contributions

WQ: Data curation, Formal analysis, Investigation, Methodology, Validation, Writing – original draft. CN: Data curation, Formal analysis, Investigation, Methodology, Writing – review & editing. HS: Data curation, Formal analysis, Investigation, Methodology, Project administration, Writing – review & editing. MC: Data curation, Project administration, Supervision, Writing – review & editing. KY: Data curation, Methodology, Project administration, Writing – review & editing. SN: Project administration, Resources, Supervision, Writing – review & editing. BY: Writing – review & editing. XH: Conceptualization, Funding acquisition, Investigation, Project administration, Resources, Supervision, Writing – review & editing.

## Supplementary material

The Supplementary material for this article can be found online at:

https://www.frontiersin.org/articles/10.3389/fnins.2024.1371319/full#supplementary-material
