# Supplementary material for: Long-term effects of mobile exoneuromusculoskeleton (ENMS)-assisted self-help telerehabilitation after stroke
Source: Front Neurosci. 2024 Mar 13;18:1371319. doi: 10.3389/fnins.2024.1371319 (PMC10965606; doi:10.3389/fnins.2024.1371319)
Supplement: Supplementary file 1 [file Table_1.DOCX]

**Supplementary Table 1** The clinical scores before, after, 3 months and 6 months after the training. The mean values and 95% confidence intervals for each measurement of the clinical assessments and the probabilities with the estimated effect sizes of the statistical analyses. The significant differences are indicated by * (*p* ≤ 0.05), ** (*p* ≤ 0.01), and *** (*p* ≤ 0.001) for one-way repeated measures ANOVA tests or Friedman tests.

| **Clinical assessments** | **Pre1** | **Pre2** | **Pre3** | **Post** | **3MFU** | **6MFU** | **One-way repeated measures ANOVA** | **Friedman test** |
| --- | --- | --- | --- | --- | --- | --- | --- | --- |
|  | **Mean value (95% confidence interval)** | | | | | | ***p* (Partial η^2^)** | ***p* (Kendall’s W)** |
| FMA-UE | 27.30(23.00~31.60) | 27.25(22.41~32.09) | 27.15(22.67~31.63) | 32.90(28.31~37.49) | 30.10(26.03~34.17) | 31.25(26.39~36.11) |  | 0.000*** (0.518) |
| FMA shoulder/elbow | 19.00(16.32~21.68) | 18.55(15.30~21.80) | 18.95(16.02~21.88) | 22.10(19.32~24.88) | 21.05(18.49~23.61) | 22.65(19.65~25.65) |  | 0.000*** (0.421) |
| FMA wrist/hand | 8.30(6.35~10.25) | 8.70(6.81~10.59) | 8.20(6.39~10.01) | 10.80(8.78~12.82) | 9.05(7.17~10.93) | 8.60(6.41~10.79) |  | 0.001*** (0.281) |
| MAS elbow | 1.79(1.51~2.07) | 1.69(1.46~1.92) | 1.76(1.52~2.00) | 1.42(1.26~1.58) | 1.57(1.41~1.73) | 1.65(1.36~1.94) |  | 0.046* (0.133) |
| MAS wrist | 1.49(1.11~1.87) | 1.40(1.10~1.70) | 1.35(1.06~1.64) | 1.09(0.88~1.30) | 1.20(0.94~1.46) | 0.95(0.69~1.21) |  | 0.001*** (0.286) |
| MAS finger | 1.81(1.45~2.17) | 1.53(1.16~1.90) | 1.63(1.23~2.03) | 1.31(1.06~1.56) | 1.47(1.18~1.76) | 1.29(0.94~1.64) |  | 0.011* (0.186) |
| ARAT | 13.75(8.22~19.28) | 13.10(7.68~18.52) | 13.30(8.13~18.47) | 17.40(12.24~22.56) | 14.50(9.85~19.15) | 14.85(9.70~20.00) |  | 0.000*** (0.406) |
| WMFT score | 33.00(26.97~39.03) | 32.65(26.77~38.53) | 33.45(27.76~39.14) | 39.55(34.47~44.63) | 37.45(32.50~42.40) | 38.30(32.70~43.90) | 0.000*** (0.438) |  |
| WMFT time | 70.28(60.47~80.10) | 70.40(60.56~80.23) | 69.82(59.28~80.36) | 63.96(53.89~74.03) | 63.31(52.85~73.76) | 57.83(46.10~69.56) |  | 0.005** (0.213) |
| FIM | 62.75(61.03~64.47) | 62.75(61.03~64.47) | 62.75(61.03~64.47) | 63.35(61.76~64.94) | 63.50(61.91~65.09) | 63.55(61.98~65.12) |  | 0.001*** (0.281) |

**Supplementary Table 2.** Normalized EMG activation level, normalized co-contraction index, NMUs and MTD before, after, 3 months and 6 months after the training. The mean values and 95% confidence intervals for each measurement of the EMG/kinematic parameters and the probabilities with the estimated effect sizes of the statistical analyses. The significant differences are indicated by * (*p* ≤ 0.05) and ** (*p* ≤ 0.01) for one-way repeated measures ANOVA test or Friedman tests.

| **EMG parameters** | **Pre** | **Post** | **3MFU** | **6MFU** | **One-way repeated ANOVA** | **Friedman test** |
| --- | --- | --- | --- | --- | --- | --- |
|  | **Mean value (95% confidence interval)** | | | | ***p* (Partial η^2^)** | ***p* (Kendall’s W)** |
| **Normalized**  **EMG activation level** |  |  |  |  |  |  |
| ECU-ED | 0.09(0.07~0.12) | 0.08(0.06~0.10) | 0.09(0.07~0.10) | 0.07(0.06~0.09) |  | 0.618(0.015) |
| APB | 0.08(0.06~0.10) | 0.08(0.05~0.10) | 0.07(0.06~0.09) | 0.05(0.04~0.06) |  | 0.088(0.055) |
| TRI | 0.06(0.05~0.08) | 0.07(0.05~0.08) | 0.06(0.05~0.07) | 0.05(0.04~0.07) |  | 0.423(0.023) |
| BIC | 0.09(0.07~0.11) | 0.07(0.05~0.08) | 0.06(0.05~0.07) | 0.06(0.05~0.08) |  | 0.221(0.037) |
| FCR-FD | 0.09(0.07~0.11) | 0.07(0.06~0.08) | 0.06(0.05~0.08) | 0.05(0.04~0.06) |  | 0.012* (0.091) |
| **Normalized**  **co-contraction index** |  |  |  |  |  |  |
| ECU-ED/APB | 0.05(0.04~0.06) | 0.04(0.03~0.05) | 0.05(0.04~0.06) | 0.04(0.03~0.05) |  | 0.024* (0.079) |
| ECU-ED/FCR-FD | 0.06(0.05~0.06) | 0.05(0.04~0.06) | 0.05(0.04~0.06) | 0.04(0.03~0.05) |  | 0.010** (0.095) |
| ECU-ED/BIC | 0.06(0.05~0.07) | 0.04(0.03~0.05) | 0.05(0.04~0.06) | 0.05(0.04~0.05) |  | 0.115(0.049) |
| ECU-ED/TRI | 0.05(0.04~0.06) | 0.05(0.04~0.06) | 0.05(0.04~0.06) | 0.04(0.03~0.05) |  | 0.695(0.012) |
| FCR-FD/APB | 0.05(0.04~0.07) | 0.05(0.04~0.05) | 0.04(0.03~0.05) | 0.03(0.02~0.04) |  | 0.018* (0.084) |
| FCR-FD/BIC | 0.05(0.04~0.06) | 0.04(0.04~0.05) | 0.04(0.03~0.05) | 0.03(0.03~0.04) |  | 0.035* (0.072) |
| FCR-FD/TRI | 0.05(0.04~0.06) | 0.05(0.04~0.06) | 0.04(0.03~0.05) | 0.03(0.03~0.04) |  | 0.252(0.034) |
| APB/BIC | 0.05(0.04~0.06) | 0.04(0.03~0.05) | 0.04(0.03~0.05) | 0.03(0.03~0.04) |  | 0.015* (0.087) |
| APB/TRI | 0.05(0.04~0.06) | 0.04(0.03~0.05) | 0.04(0.03~0.05) | 0.03(0.02~0.04) |  | 0.466(0.021) |
| BIC/TRI | 0.05(0.04~0.06) | 0.04(0.03~0.05) | 0.04(0.03~0.05) | 0.04(0.03~0.05) |  | 0.927(0.004) |
| **Kinematic parameters** |  |  |  |  |  |  |
| NMUs | 32.77(27.75~37.78) | 24.50(20.72~28.28) | 29.40(25.04~33.77) | 28.08(23.72~32.45) |  | 0.010** (0.095) |
| MTD | 177.70(158.27~197.12) | 178.63(160.47~196.78) | 161.89(142.53~181.25) | 159.87(139.26~180.49) | 0.005** (0.104) |  |
